# 泽布替尼治疗B细胞肿瘤患者中枢神经系统侵袭性真菌病3例报告并文献复习

**DOI:** 10.3760/cma.j.cn121090-20241129-00495

**Published:** 2025-06

**Authors:** 振硕 金, 月华 黄, 凡 于, 毅 郭, 生 董, 利红 李, 燕婴 王

**Affiliations:** 1 清华大学附属北京清华长庚医院血液内科，清华大学临床医学院，北京 102218 Department of Hematology, Beijing Tsinghua Changgung Hospital, School of Clinical Medicine, Tsinghua University, Beijing 102218, China; 2 清华大学附属北京清华长庚医院神经外科，清华大学临床医学院，北京 102218 Department of Neurosurgery, Beijing Tsinghua Changgung Hospital, School of Clinical Medicine, Tsinghua University, Beijing 102218, China

## Abstract

布鲁顿酪氨酸激酶抑制剂（BTKi）的出现为B细胞肿瘤患者提供了更多治疗选择，但近年来随着其广泛应用，患者发生感染的风险增加。本文报道3例B细胞肿瘤患者，包括1例慢性淋巴细胞白血病和2例弥漫大B细胞淋巴瘤患者在接受BTKi泽布替尼单药或联合治疗期间出现中枢神经系统侵袭性真菌病（IFD）事件，所有患者接受抗真菌治疗均有效，达到完全缓解；中位随访35个月，所有患者均为持续缓解状态且IFD均未复发。

## Abstract (en)

The advent of bruton tyrosine kinase inhibitors（BTKi）has offered more therapeutic choices for patients with B-cell malignancies. However, with its widespread application in recent years, the use of BTKi increases the risk of severe infections in patients. Here, we report three cases of B-cell malignancies, including one case of chronic lymphocytic leukemia and two cases of diffuse large B-cell lymphoma. During treatment with BTKi zanubrutinib, different degrees of invasive fungal disease（IFD）of the central nervous system occurred in these patients. All patients fully recovered following antifungal therapy. With a median follow-up of 35 months, all patients remained in a continuous remission state, and none of them had a recurrence of IFD.

布鲁顿酪氨酸激酶抑制剂（BTKi）是治疗B细胞肿瘤的新型靶向药物，伊布替尼为第一代BTKi，由于其存在脱靶效应[1]，感染、心房颤动等治疗相关不良事件时有报道[2]。泽布替尼是新一代BTKi，对BTK的特异性较高，能最大限度地减少脱靶效应和相关不良反应的发生风险，具有更高的生物利用度及持续治疗能力[3]。目前真实世界中关于泽布替尼治疗期间出现侵袭性真菌病（IFD）的研究较少，特别是中枢神经系统IFD。本文描述了3例B细胞肿瘤患者接受泽布替尼单药或联合治疗期间出现中枢神经系统IFD的诊疗过程并进行相关文献复习。

## 病例资料

根据《血液病/恶性肿瘤患者侵袭性真菌病的诊断标准与治疗原则（第六次修订版）》[4]确定了3例接受泽布替尼单药或联合治疗后确诊为IFD的患者。

例1，女，77岁，慢性淋巴细胞白血病（CLL），Rai Ⅲ期，Binet C期，CLL-IPI评分为4分、高危，中度贫血（HGB 76 g/L）。行泽布替尼（160 mg，每日2次）单药治疗，2个月后评估疗效达到骨髓未恢复的完全缓解（CRi）。2.5个月时出现头痛、左侧肢体无力伴行走不稳。查体：左侧肢体肌力4级、针刺觉减退、巴宾斯基征阳性。头颅增强核磁共振（MRI）提示右侧额顶叶皮层及皮层下见多发结节状长短混杂T2信号，可见囊腔，病灶周围见指状水肿包绕（图1）。对右侧顶叶病灶行立体定向脑活检及抽吸囊液，脑组织病理提示增生的纤维组织内见组织细胞、淋巴细胞及中性粒细胞浸润，并见散在多核巨细胞，小血管增生扩张充血伴出血，见大片坏死组织。PAS及六胺银染色均阴性。囊液镜检及培养均阴性，宏基因组学二代测序（NGS）提示烟曲霉（序列数22）。给予伏立康唑治疗3个月，1周后患者头痛明显缓解，左上肢肌力恢复、左下肢肌力4级；1个月后患者所有症状均消失；3个月后复查头颅MRI提示右额叶和顶叶病变范围较前明显减小，水肿明显好转，疗效达到部分缓解（PR）。停用泽布替尼，改为苯丁酸氮芥（2 mg，每天3次）治疗，随访35个月，患者持续CRi状态，IFD未复发。

**图1:**
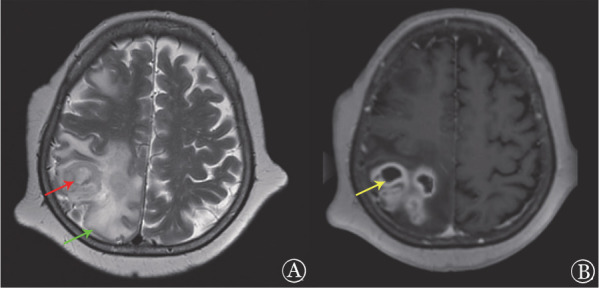
慢性淋巴细胞白血病患者（例1）发生中枢神经系统侵袭性真菌病的头颅增强核磁共振（MRI）表现 **A** MRI轴位T2，右侧额顶叶皮层及皮层下见多发结节状长短混杂T2信号，增强扫描呈变化强化（红色箭头），病灶周围见指状水肿包绕（绿色箭头）；**B** MRI轴位T1，可见囊腔，囊壁厚薄均匀，未见明确壁结节样强化（黄色箭头）

例2，女，61岁，弥漫大B细胞淋巴瘤，非生发中心型（DLBCL, non-GCB），BCL2和MYC双表达，Lugano分期Ⅲ期A组，IPI评分3分、中高危。行ZR-CHOP（泽布替尼+利妥昔单抗+环磷酰胺+表阿霉素+长春地辛+泼尼松）方案治疗，4个周期后评估疗效为代谢学部分缓解（PMR）。第5个周期治疗开始前3 d出现发热，最高体温39.5 °C，伴剧烈头痛、咳嗽、喘憋；症状发生3周前有鸟类密切接触史。查体：四肢肌力3～4级、双侧病理征阴性、脑膜刺激征阴性。外周血NGS提示新型隐球菌（序列数5）。头颅MRI未见显著异常；胸部CT提示双肺可见多发磨玻璃密度影、双侧胸腔少量液性密度影（图2A）。电子气管镜检查提示气管-支气管树急性炎症改变（左侧为著），支气管肺泡灌洗液提示新型隐球菌抗原阳性。脑脊液常规未见异常，蛋白阴性、葡萄糖4.38 mmol/L（正常范围：2.22～3.89 mmol/L）、氯化物118 mmol/L（正常范围：120～132 mmol/L），脑脊液镜检及培养阴性，新型隐球菌抗原阳性，NGS提示新型隐球菌（序列数399）。给予两性霉素B、氟康唑诱导治疗，3 d后患者体温恢复，1周后头痛、咳嗽缓解；2周后胸部CT提示双肺多发磨玻璃密度影较前明显吸收，脑脊液的葡萄糖及氯化物水平恢复正常；6周后胸部CT提示病灶完全吸收，疗效达到完全缓解（图2B），继续氟康唑巩固及维持治疗共1年。停用泽布替尼，调整为R-GDP（利妥昔单抗+吉西他滨+顺铂+泼尼松）方案应用2个周期，终末评估达代谢学完全缓解（CMR），随访48个月，患者持续CMR状态，IFD未复发。

**图2:**
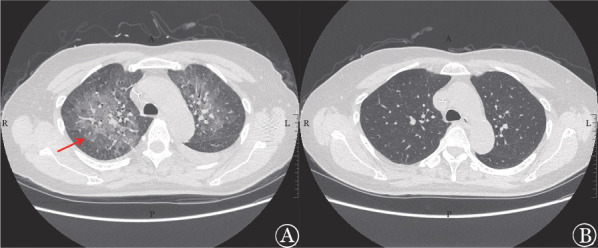
弥漫大B细胞淋巴瘤患者（例2）隐球菌肺炎的胸部CT表现 **A** 出现症状时胸部CT：双肺可见多发磨玻璃密度影，呈铺路石样改变，上叶为著（红色箭头），双侧胸腔少量液性密度影；**B** 治疗6周后胸部CT：磨玻璃影已完全吸收，未见液性密度影

例3，女，75岁，DLBCL, non-GCB，BCL2和MYC双表达，Lugano分期Ⅳ期B组，IPI评分4分、高危。行1个周期R-DA-EPOCH（利妥昔单抗+依托泊苷+泼尼松+长春地辛+环磷酰胺+表阿霉素）方案后出现粒细胞缺乏症、革兰阴性杆菌败血症，后改为ZR-CHOP方案化疗，中期评估疗效达CMR。第5个周期化疗后出现胸闷、双下肢无力；查体：右下肺触觉语颤增强、可闻及少许干啰音，左下肢肌力4级、右下肢肌力3级。胸部CT提示右肺上叶胸膜下见团片影，与胸膜分界不清；头颅MRI未见显著异常，椎体MRI提示第4胸椎体、附件及椎管内可见短T1、长短混杂T2信号，继发相应节段椎管狭窄，脊髓受压向后移位（图3）。行CT引导胸部病灶穿刺，病理提示右肺上叶病变：少许穿刺坏死肺组织、肺泡腔及血管腔残影中见真菌菌丝残影，考虑为毛霉菌，特殊染色：PAS（+）。行局部椎体病灶清除术、椎管减压术及椎体植骨融合内固定术，术中见肌肉间隙内黄白色黏稠脓液，延伸至第4胸椎右侧神经根腹侧硬膜外；病理提示椎管内占位性病变：神经纤维组织伴坏死，其中散在可见淋巴细胞及组织细胞浸润；椎旁肌肉内脓肿组织：炎性肉芽组织，出血，脓肿形成，伴钙化及多核巨细胞反应，纤维组织增生，局部可见少许菌丝，菌丝较粗，考虑为毛霉菌。给予两性霉素B脂质体治疗，2周后患者症状明显减轻，3周后肌力恢复，4周后复查胸部CT仍可见右肺团块样病灶，胸椎MRI提示第4胸椎变扁、内部信号不均，可见金属植入物，第3至第5椎体部分附件术后缺如，考虑患者达到PR疗效，改为泊沙康唑治疗，持续半年。停用泽布替尼，调整为R-CHOP方案应用1个周期，终末评估CMR，随访33个月，患者持续CMR状态，IFD未复发。

**图3:**
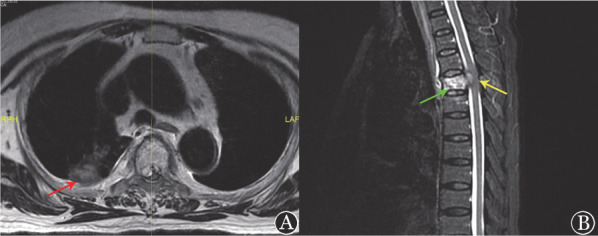
弥漫大B细胞淋巴瘤患者（例3）发生侵袭性真菌病的椎体增强核磁共振（MRI）表现 **A** 胸椎MRI轴位T2，右肺上叶后段胸膜下团块状长T2信号（红色箭头）；**B** 胸椎MRI压脂矢状位，第4胸椎椎体、附件及椎管内可见短T1、长短混杂T2信号（绿色箭头），继发相应节段椎管狭窄（黄色箭头），脊髓受压向后移位

## 讨论并文献复习

BTK是B细胞表面受体（BCR）介导的信号通路关键组成部分，在B细胞肿瘤的发生中起重要作用[5]，因此BTKi的成功研发，为治疗B细胞肿瘤带来了突破性进展[6]，但随之而来的是感染发生率的增加[7]–[9]。临床研究证明，伊布替尼治疗与患者真菌感染风险增加相关[10]。一项以伊布替尼为基础治疗原发性中枢神经系统淋巴瘤（PCNSL）的试验中，39％患者出现侵袭性曲霉菌病、11％患者出现肺孢子菌肺炎（PCP）[11]。法国一项回顾性研究[7]发现，接受伊布替尼治疗B细胞肿瘤的患者中，33例发生IFD，40.7％累及中枢神经系统。

中性粒细胞和巨噬细胞是人类抵抗真菌感染的重要防线[12]。BTK功能缺失的小鼠在中性粒细胞的表达方面存在缺陷且对炎症刺激的反应较差[13]–[14]。BTK通过作用于E-选择素而增加中性粒细胞的募集、参与中性粒细胞对真菌的识别、释放颗粒蛋白和活性氧以及吞噬较小的孢子囊等控制真菌感染[15]–[16]。因此，BTKi一定程度上会抑制中性粒细胞的功能，从而降低对真菌感染的防御能力。Dectin-1是巨噬细胞主要的识别受体，与真菌β-葡聚糖相互作用；BTK可以通过活化T细胞核因子（NFAT）转录因子的核定位并激活NF-κB途径，从而参与Dectin-1识别受体激活[16]。小鼠实验表明伊布替尼可抑制巨噬细胞NFAT及NF-κB的激活从而减弱巨噬细胞对曲霉菌的清除作用[17]。BTKi还可通过影响NLRP3炎症小体的激活来调节巨噬细胞的炎症反应[18]。此外BTKi可抑制T细胞的激活、降低免疫球蛋白水平，从而进一步增加真菌感染易感性[19]。在伊布替尼治疗期间，感染一般发生在前6个月内，侵袭性真菌感染的中位发生时间为开始治疗的前4个月[5],[20]–[22]。

泽布替尼为第2代共价结合型高选择性BTKi，真菌感染事件也时有报道。泽布替尼单药治疗复发/难治套细胞淋巴瘤Ⅱ期临床研究的3年随访发现86例患者中有3例疑似发生IFD[23]；华氏巨球蛋白血症的3年随访发现50例患者中有3例发生IFD[24]；初治及复发难治CLL患者的长期随访研究发现，3例患者发生隐球菌感染，1例发生曲霉菌感染[25]。国外一项关于泽布替尼单药治疗B细胞肿瘤患者的安全性分析发现泽布替尼与其他BTKi感染发生率相似[26]，一项基于食品药品监督管理局不良事件报告系统（FAERS）数据库关于泽布替尼在真实世界的安全性研究发现，泽布替尼与真菌性肺炎的相关性是数据库中其他药物的23.7倍，隐球菌性脑膜炎为40.1倍，PCP为15.5倍[27]。以上研究均表明泽布替尼相较于伊布替尼，IFD的发生率并无显著差别[28]。

本文报道了3例在接受泽布替尼治疗期间出现中枢神经系统IFD的B细胞肿瘤患者，均在开始治疗后3个月内发生，与伊布替尼致IFD中位时间相同，表明二者发生真菌感染可能有着相似的机制。除了应用BTKi外，血液系统恶性肿瘤患者发生IFD的危险因素还包括应用糖皮质激素、粒细胞缺乏、严重营养不良等[4],[9],[29]。本文中3例患者除应用BTKi外，还同时存在多种危险因素，例1合并低丙种球蛋白血症、严重营养不良，例2、3合并应用糖皮质激素以及细胞毒性药物，并出现粒细胞缺乏。此外例2曾在感染发生前1个月密切接触鸟类。同时，本文3例患者中2例合并真菌性肺炎，特别是例3，影像学提示肺内病灶与椎体、椎管内病灶相邻，因此高度怀疑其椎管内毛霉菌感染为肺内病灶侵袭播散所致。

根据《血液肿瘤免疫及靶向药物治疗相关性感染预防及诊治中国专家共识（2021年版）》[30]，对BTKi治疗相关IFD应重点监测，以便早期发现。我们对3例接受泽布替尼治疗后发生中枢神经系统IFD的B细胞肿瘤患者进行长期随访，在停用泽布替尼并规律行抗真菌治疗后，患者感染可被有效控制，并在长期随访中未再出现中枢神经系统真菌感染事件。由此推测，泽布替尼会增加真菌感染的风险；对于已经开始接受BTKi治疗的B细胞肿瘤患者，应及时评估同时存在的其他危险因素，及早采取针对性措施，有可能避免或减少中枢神经系统IFD的发生。
